# Single-Drop Analysis of Epinephrine and Uric Acid on a Screen-Printed Carbon Electrode

**DOI:** 10.3390/bios11080285

**Published:** 2021-08-19

**Authors:** David Majer, Matjaž Finšgar

**Affiliations:** Faculty of Chemistry and Chemical Engineering, University of Maribor, Smetanova ulica 17, 2000 Maribor, Slovenia; david.majer@um.si

**Keywords:** epinephrine, uric acid, single-drop analysis, screen-printed electrode, weighted linear regression

## Abstract

This work demonstrates the analysis of epinephrine (EP) and uric acid (UA) in a single drop (the volume of the test solution was only 50 µL) using a screen-printed carbon electrode (SPCE) sensor and square-wave voltammetry (SWV). The limit of detection, limit of quantification, linearity, accuracy, precision, and robustness were validated. The normality of the experimental data was tested and confirmed for both methods. Heteroscedasticity was checked by residual analysis followed by a statistical *F*-test. The latter was confirmed for both analytes. The low relative standard deviations (RSD) at all calibration points and repetitive slopes justified the use of a calibration curve; therefore, the standard addition methodology was avoided (the latter is common in electroanalysis, but time-consuming). Since the conditions for using an ordinary least squares (OLS) regression were not met, weighted linear regression (WLR) was used to improve the accuracy of the analytical results at low concentrations of the analytes. In this manner, the best weighted model was determined and used for the quantification. A comparison was made between the OLS and WLR methods to show the necessity of using the WLR method for EP and UA analysis. The newly developed and validated methods were also shown to be effective in the analysis of real samples. The content of EP in an EP auto-injector and UA in human urine was tested by employing the best weighted model. For EP and UA, the accuracy in terms of the average recovery value was 101.01% and 94.35%, and precision in terms of RSD was 5.65% and 2.75%, respectively. A new analytical methodology is presented that uses a low volume (a single drop), and it offers the advantage of electroanalysis for on-site analysis, where conventional chromatographic techniques cannot be easily employed. Furthermore, the developed technique has additional advantages in terms of speed, cost, and miniaturization.

## 1. Introduction

Epinephrine (EP) (also named adrenaline) is an important and well-known member of the catecholamine family produced by the adrenal glands; it is also present in low concentrations in extra-adrenal tissues, primarily in sympathetic nerves. It is classified as a hormone and neurotransmitter in the mammalian central nervous system [1,2,3]. Many physiological phenomena are related to the concentration of EP in blood [4,5]. EP can also be used as a drug and is used in the treatment of cardiac surgery, bronchial asthma, hypertension, cardiac arrest, asthma, heart blockages, anaphylaxis, etc. [6,7,8]. EP constricts blood vessels, and thus increases the heart rate, dilates airways, and is known to trigger the flight-or-fight response of the sympathetic nervous system [9,10]. Anaphylaxis is a potentially fatal allergic reaction involving multiple organ systems that can lead to respiratory compromise, hypotension, and even death. For the treatment of anaphylactic shock, EP is used in the form of an easy-to-use auto-injector due to its cardiovascular and bronchial effects [11,12,13].

Uric acid (UA) is the end product of the metabolic degradation of purine nucleotides, such as guanine and adenine, and is important for renal and cardiovascular function [14,15]. It can be found in human body fluids such as blood and urine [16]. Monitoring UA is important because abnormal levels of UA can lead to gout, hyperuricemia, and Lesch–Nyhan syndrome [17,18,19,20]. In addition, elevated levels of UA have been associated with pneumonia and leukemia [21,22].

Analysis of EP and UA can be performed using high-performance liquid chromatography [23,24], gas chromatography [25,26], spectrophotometry [27,28], capillary electrophoresis [29,30,31], and electroanalytical methods [32,33,34,35,36]. The advantages of electroanalytical methods for EP and UA determination, compared to the above techniques, are high sensitivity, the low cost of analysis and instrumentation, and a fast analysis time [22,37]. One aspect of electroanalytical instrumentation that has developed rapidly in recent years is screen-printed electrodes (SPEs). The array of electrodes on an SPE consists of a working electrode, a reference electrode, and a counter electrode printed on the same substrate surface by the screen-printing technique. The screen-printing technique is a well-established technique for fabricating low-cost, portable, and disposable electrode systems. In addition to its low-cost effectiveness and portability, the advantages of SPEs over a conventional three-electrode cell system are miniaturization, the ability to analyze a small sample volume, disposability, and on-site and real-time analysis [38,39]. An important example of the commercialization of SPEs is the biosensor for glucose monitoring used in patients with diabetes [40]. The working electrode of an SPE is frequently made of conductive inks based on platinum, gold, silver, or carbon. On a screen-printed carbon electrode (SPCE), the working electrode is made of carbon, which is the most commonly used material due to its versatility and low cost [41]. An electrochemical analysis is usually performed by immersing a conventional three-electrode system or the SPE in an electrochemical cell containing a solution of analyte(s), the supporting electrolyte, and a stir bar [42]. The alternative option, which has not been widely reported or investigated, is single-drop analysis of the sample. In this case, a small volume of sample (e.g., a volume of 50 µL, one drop) is pipetted on to the surface of the SPE so that all three electrodes (working, reference, and counter electrodes) are covered. Single-drop analysis simplifies on-site analysis as no additional electrochemical cell or stirrer is required, and just a smartphone and handheld potentiostat suffice [43]. In addition, single-drop analysis can be used when only a small sample volume (e.g., 50 µL) is available for analysis.

Only a few reports have been published on the determination of analytes using single-drop analysis. For example, Tseliou et al. [44] developed a lab-on-a-screen-printed sensor for the detection of flunitrazepam, which is known as a date-rape drug. Direct drop-volume application for untreated and undiluted samples was demonstrated. In another study, Stankovič et al. [45] reported the determination of the cytostatic drug doxorubicin in biological fluids and in a pharmaceutical product using a screen-printed diamond electrode. The analysis was performed using a single drop of solution. A single-drop analysis was also used by Couto et al. [46] in a study in which SPCE based on a molecularly imprinted polymer was developed for the selective determination of 3,4-methylenedioxymethamphetamine (MDMA) in biological samples.

Validation of an analytical method is a process of defining an analytical requirement and proving that the analytical method under consideration has capabilities consistent with what a given application requires [47]. The validity of analytical methods depends on the guidelines, terminology, and methodology proposed in the applicable documents. However, there may be several differences between these documents, so more than one document is usually used [48,49]. When validating an analytical method, it is essential to determine the range in which the instrumental response (i.e., measured signal) is proportional to the concentration of the analyte. This relationship is most commonly determined using linear regression, which estimates the regression parameters using the ordinary least squares (OLS) method. The OLS method assumes that variances (*s*^2^) in the response are not statistically significantly different (i.e., the *s*^2^ are statistically equal at every calibration point) [50]. If the values of *s*^2^ are statistically different for different calibration points (i.e., heteroscedasticity) and increase with concentration, weighted linear regression (WLR) should be used instead of OLS. Otherwise, a significant loss in accuracy and precision, especially at a lower linear calibration range, can be expected [51,52].

This work presents the development and validation of methods for the determination of EP and UA in real samples using a single drop (50 µL) placed on the surface of an SPCE. The WLR method was used to improve the calibration model since the heteroscedasticity of the measured data was observed for both analytes. Single-drop analysis combined with such statistical analysis provides an accurate, precise, and rapid method for the individual determination of EP and UA in real samples, which, to the best of the authors’ knowledge, has not been reported previously.

## 2. Experimental Methods

All electrochemical measurements in this work were performed with a PalmSens4 potentiostat/galvanostat under laboratory conditions (23 ± 2 °C), supplied by PalmSens (Houten, The Netherlands). The PalmSens4 was controlled using PSTrace 5.8 software.

### 2.1. Solutions and Reagents

Na_2_HPO_4_ ∙ 7H_2_O (purity > 99%) and NaH_2_PO_4_ ∙ H_2_O (purity > 98%) were supplied by Acros Organics (Fair Lawn, NJ, USA). Potassium hexacyanoferrate(III) (99%) (K_3_Fe[CN]_6_) and UA (purity ≥ 99%) were supplied by Sigma Aldrich (St. Louis, MO, USA). USP standard EP bitartrate was supplied by Sigma Aldrich (Rockville, MD, USA). KCl and HCl (37%, for analysis-ISO) were supplied by Carlo Erba Reagents (Val de Reuil, France). All solutions of EP and UA standards were prepared in 0.15 M (pH = 6.5) phosphate buffer solution (PBS), which served as the supporting electrolyte. The PBS was prepared using ultrapure water with a resistivity of 18.2 MΩ cm, obtained using the ELGA (Lane End, UK) water purification system.

### 2.2. Screen-Printed Electrodes

SPE sensors, model AC1.W4.R2, were supplied by BVT Technologies (Brno, Czech Republic) and used as a three-electrode electrochemical system. These SPE sensors have a working electrode (WE) and counter electrode (CE) made of carbon (the diameter of the WE was 1 mm), while the reference electrode (RE) was made of Ag that was oxidized to AgCl (by the supplier). All potentials (*E*) in this work are reported vs. this Ag/AgCl reference electrode.

To test the robustness of an analytical method, SPE sensors from a different manufacturer were also employed. These SPEs (model DRP-110) were supplied by DropSens (Llanera, Spain). These sensors had a WE with a diameter of 4 mm. Both the WE and CE were made of carbon, while the reference electrode was made of Ag. Hereinafter, the term SPCE is used for AC1.W4.R2 SPEs, supplied by BVT Technologies, since the WE was made of carbon. The term D-SPCE is used hereinafter in the case of SPEs supplied by DropSens (type DRP-110).

For each new SPE sensor employed, the surface was first cleaned using a drop of 0.1 M HCl that was placed on to the surface of an SPCE (or D-SPCE) sensor and an *E* of 1.000 V was applied for 5 min. This chemical/electrochemical cleaning procedure was employed to remove any possible contaminants from the electrode’s surface. Afterwards, the surface was rinsed with ultrapure water and dried.

### 2.3. Square-Wave Voltammetry

Square-wave voltammetry (SWV) was used for method validation and real sample analysis due to its high sensitivity and short analysis time [53]. SWV measurements started at an *E* of −0.600 V. An *E* sweep was then performed in the anodic direction until a final *E* of 1.000 V was reached. An *E* step of 4 mV, an amplitude of 50 mV, and a frequency of 20 Hz were employed. Once a drop of solution was placed on the surface of an SPCE, the SWV voltammogram was measured. The SWV voltammogram was obtained in 20 s.

### 2.4. Single-Drop Analysis on an SPCE Sensor

Single-drop analysis on the surface of an SPCE (or D-SPCE) sensor using SWV was performed as follows. Separate solutions of standards of different UA or EP concentrations required for validation and analysis were prepared. For analysis, a 50 µL drop was pipetted on to the surface of an SPCE sensor so that all three electrodes were covered. Immediately after applying the drop, a measurement was carried out. To obtain good analytical results, only one voltammogram measurement should be performed. Two or more consecutive voltammogram measurements with the same drop of solution became problematic, as the current decreased when a second measurement using the same drop of solution was performed. A decrease in current was also observed when a small volume of solution of analyte standard was pipetted directly on to a pre-existing drop on the surface, making such analysis analytically non-useful. On the other hand, an analytically favorable procedure was obtained and employed, as presented hereinafter, when after each measurement, the surface of the SPCE was rinsed with ultrapure water and the remaining water was soaked up with a paper towel without touching the active WE surface. Then, the surface was dried under a stream of N_2_ gas. After this cleaning procedure, another drop was pipetted, and a new measurement was performed.

### 2.5. Cyclic Voltammetry

Cyclic voltammetry (CV) measurements were performed every day before analysis to ascertain the suitability of the SPCE and D-SPCE sensors. The suitability of both sensors was checked using K_3_Fe[CN]_6_, which is known to be diffusion-controlled and reversibly oxidized, and reduced on the surface of the WE [54]. Measurements were carried out in a 1.0 M KCl solution containing 10 mM K_3_Fe[CN]_6_. CV measurement began at a starting *E* of 0.800 V. The *E* was then swept in the cathodic direction until a switching *E* of −0.300 V was reached. The *E* sweep was then reversed in the anodic direction until a final *E* of 0.800 V was reached. A step of 4 mM was employed. To verify the reversible and diffusion-controlled reaction of K_3_Fe[CN]_6_, scan rates (*ν*) of 10, 20, 50, 75, 125, 150, 175, and 200 mV/s were employed. To start a CV experiment, a 50 µL drop of 10 mM K_3_Fe[CN]_6_ in 0.1 M KCl solution was pipetted on to the surface of the sensor so that all three electrodes were covered. The cyclic voltammogram was then measured using a *ν* of 10 mV/s. The sensor was then rinsed with ultrapure water, the remaining ultrapure water was soaked up with a paper towel without touching the active WE surface, and the sensor was dried under a stream of N_2_ gas. Another drop of 10 mM K_3_Fe[CN]_6_ in 0.1 M KCl solution was then pipetted on to the surface of the sensor and a CV measurement was performed using a *ν* of 20 mV/s. This procedure was repeated with increasing *ν* until a *ν* of 200 mV/s was reached. Both sensors had to meet several criteria for the reversible and diffusion-controlled reaction to be used later for further analysis [55]. The criteria and results are given in the Supplementary Material (see Figure S1). Whenever large deviations from the criteria were observed, a sensor was discarded and replaced with a new one.

### 2.6. Real Sample Analysis

For real sample analysis, the content of EP in an auto-injector and the content of UA in human urine were tested. EP from an auto-injector was diluted with 0.15 M PBS solution before analysis. To determine the amount of UA in human urine, a sample of human urine was collected from one of our colleagues and, without any pre-treatment, diluted with 0.15 M PBS solution before analysis. A volume of 50 µL for both real samples was employed for analysis. All measurements were performed in triplicate. In order to test the accuracy and precision, the real samples were spiked with a known amount of solution of the corresponding analyte standard.

## 3. Results and Discussion

### 3.1. Limit of Detection and Limit of Quantification

**Figure 1:**
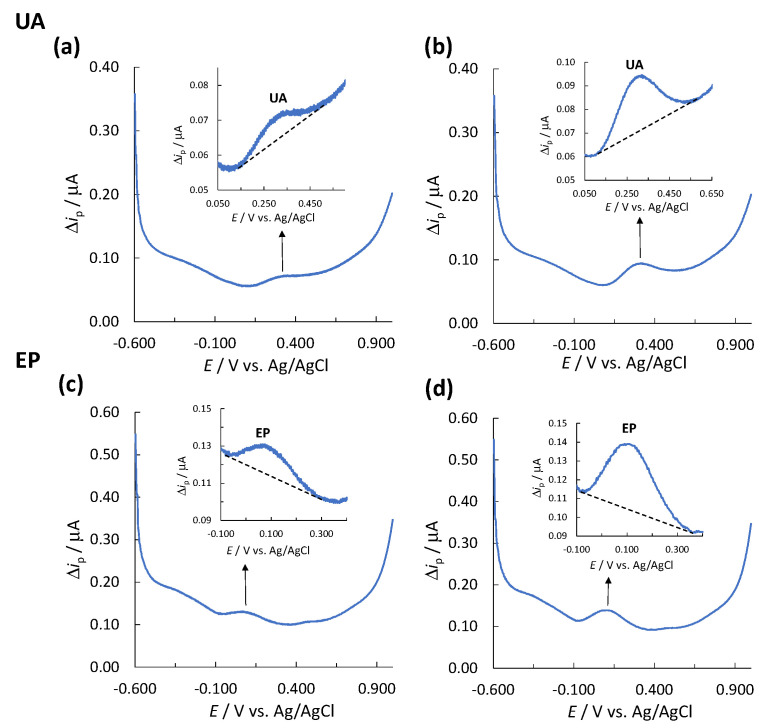
A corresponding voltammogram for the determination of (**a**) LOD for UA, (**b**) LOQ for UA, (**c**) LOD for EP, and (**d**) LOQ for EP.

Since the SWV technique exhibits a baseline (together with its baseline noise), the limit of detection (LOD) and limit of quantification (LOQ) were determined experimentally based on the signal-to-noise (S/N) ratio (signal (S) is the peak height for the analyte measured, Δ*i*_p_, and noise (N) is the difference between the largest and smallest measured current points of the baseline). In order to determine the LOD and LOQ, different concentrations of solutions of analyte standard were prepared and measured separately using SWV and single-drop analysis. S/N ratios of ≥3.00 and ≥10.00 were considered acceptable for estimation of the LOD and LOQ, respectively [56]. Three replicates of the LOD and LOQ determination were performed, and the highest values out of these measurements are reported as the values for LOD and LOQ. For UA, the determined LOD was 1.00 mg/L (S/N = 3.87) (Figure 1a) and the LOQ was 2.26 mg/L (S/N = 12.52) (Figure 1b). For EP, the determined LOD and LOQ were 1.20 (S/N = 6.24) (Figure 1c) and 2.01 mg/L (S/N = 11.55) (Figure 1d), respectively.

### 3.2. Determination of the Linear Concentration Range

#### 3.2.1. OLS Method

**Figure 2:**
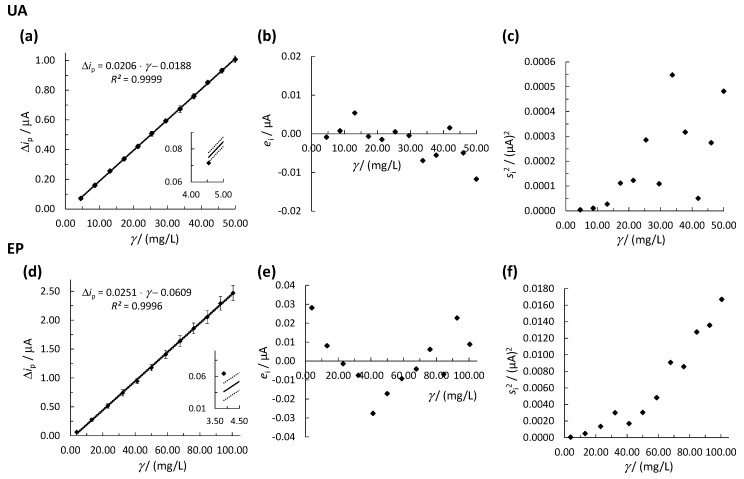
Linear concentration ranges for (**a**) UA and (**d**) EP using the OLS method, where the solid line represents the calibration curve and the dotted lines represent the upper and lower 95% confidence intervals. The error bars in (**a**,**d**) represent the standard deviations. The inserts in (**a**,**d**) show the first calibration point. Plots of the residual analysis are shown in (**b**) for UA and (**e**) for EP. Plots of $s_{i}^{2}$ vs. *γ* are shown in (**c**) for UA and in (**f**) for EP. Measurements were performed by SPCE.

The linear concentration ranges of the methods (for EP and UA determination) were determined by analysis of the prepared separate diluted solutions at different concentrations of the EP and UA standards, and the measurements were carried out using SWV and single-drop analysis, as described in Section 2.4. The relationship between Δ*i*_p_ and *γ* (where *γ* is the analyte’s mass concentration in mg/L) was evaluated first by the OLS method. Initially, the OLS method was used, and the square of the correlation coefficient (*R*^2^) value was determined for a given linear concentration range, which had to be higher than 0.9900 to accept the linearity in a given concentration range. The results for the determination of UA and EP using the OLS method are presented in Figure 2. In addition, the suitability of the calibration curves was also evaluated by the quality coefficient (QC) test [57].

Three different characteristic requirements justify the use of the OLS method: (i) all errors occur only in the *y*-direction, (ii) the errors in the *y*-direction are normally distributed, and (iii) the variation in the errors in the *y*-direction is constant for all *x*-values [58]. The SWV measurements fulfilled requirement (i) because the errors in the prepared solution concentrations were non-significant compared to the errors of the SWV signal (Δ*i*). The normal distribution of errors in the *y*-direction was checked and confirmed by visual inspection using the quantile-quantile (Q-Q) plot and by the Kolmogorov–Smirnov (K-S) statistical test to verify requirement (ii). Requirement (iii) states that the values of *s*^2^ for the replicate Δ*i* signal measurements at every calibration point ($s_{i}^{2}$) must be statistically equal over the entire calibration range. The latter checks for the required homoscedastic distribution of the experimental data in the OLS method. The heteroscedasticity of the experimental data was additionally checked by visual inspection using a residual analysis plot (Figure 2b,e) followed by the statistical *F*-test. To perform the heteroscedasticity study, three measurements were carried out at every calibration point, and the calibration curve was constructed using the average signal at every calibration point (Figure 2a,d). The experimental data were checked for possible outliers at every calibration point using Dixon’s and Grubb’s statistical tests [59]; however, no outlier was detected. The residual analysis was performed by calculating the residuals (*e*_i_) using Equation (1).

(1) $$e_{i}={\left(∆i_{p}^{\mathrm{experimental}}\right)}_{i}-{\left(∆i_{p}^{\mathrm{model}}\right)}_{i}$$

where ${\left(∆i_{p}^{\mathrm{experimental}}\right)}_{i}$ is the measured peak height current of the ith calibration point and ${\left(∆i_{p}^{\mathrm{model}}\right)}_{i}$ is the corresponding current obtained from the regression equation using the OLS method. A non-random distribution of the *e*_i_ around the *γ* axis would indicate heteroscedastic behavior of the experimental data [52]. Since the residual analysis is only a visual inspection, an additional statistical test is required [57]; therefore, a statistical *F*-test was employed. To test whether the difference between $s_{i}^{2}$ was significant, experimental *F* factor, i.e., *F*^exp^, was calculated using Equation (2).

(2) $$F^{\exp}=\frac{s_{\max}^{2}}{s_{\min}^{2}}$$

where $s_{\max}^{2}$ and $s_{\min}^{2}$ are the highest and lowest $s_{i}^{2}$, respectively. The calculated *F*^exp^ is subsequently compared with the critical value *F*^crit^ at a 95.00% confidence level for (*k* − 1) degrees of freedom, where *k* is the number of measurements at every calibration point. Heteroscedasticity is confirmed when *F*^exp^ is higher than *F*^crit^ [60,61].

The obtained linear concentration ranges for UA and EP were 4.55–50.00 mg/L (Figure 2a) and 3.87–100.49 mg/L (Figure 2d), respectively. The upper limit of the linear concentration range for UA is restricted by the limited solubility of UA in water. Both methods had *R*^2^ > 0.9900 (see Figure 2a,d) with QC < 5.00% (the calculated QC values for UA and EP were 1.54% and 0.96%, respectively), justifying the reported linear concentration ranges using the OLS method. On the other hand, the inserts in Figure 2a,d show that the first calibration point for both methods was not within the required 95% confidence interval, which, if taken into account, would lead to lower accuracy at low concentrations of the analytes. The latter calls for non-OLR regression (as explained below), even though *R*^2^ > 0.9900 for both methods. The normal distribution of the experimental data was confirmed for both cases (see Figure S2 in the Supplementary Material); therefore, requirement (ii) was fulfilled. Moreover, the residual analysis for UA in Figure 2b shows a non-random distribution of *e*_i_ around the *γ*-axis, as four out of twelve *e*_i_ appear on the positive side of the *γ* axis. A similar result is observed for EP, where five out of twelve *e*_i_ lay on the positive side of the *γ*-axis and *e*_i_ have a v-shaped distribution (but not random, as required, Figure 2e). The non-random distribution of *e*_i_ in both methods indicates the heteroscedasticity of the data. Another indicator of the statistically significantly different $s_{i}^{2}$ values across the whole calibration range can be seen in Figure 2c,f, where $s_{i}^{2}$ increase with *γ* for both methods, again confirming heteroscedasticity. The latter was also confirmed with a statistical *F*-test. The calculated *F*^exp^ for UA and EP were 135.68 and 289.27, respectively. Both values are greater than *F*^crit^ = 39.00 (*F*^crit^ was obtained at a 95.00% confidence level), indicating that there is a significant difference between $s_{i}^{2}$; thus, heteroscedasticity was confirmed. Since requirement (iii) is not met when using the OLS method to construct the linear calibration curve, the WLR method must be used instead.

#### 3.2.2. WLR Method

The WLR method is an extension of the OLS method using weights, and it is employed when the homoscedastic requirement for analytical data is not met. It works by including additional non-negative constants or weighting factors (*w*_i_) associated with each calibration point in the equations used to calculate slope (*b*_1_), intercept (*b*_0_), and *R*^2^ [62]. Higher $s_{i}^{2}$ present at higher concentrations tend to affect the regression curve more than lower $s_{i}^{2}$ at lower concentrations; hence, the determined concentration accuracy at the lower limit of the calibration curve can be significantly affected. The WLR method addresses this problem by assigning larger weights to smaller concentrations and smaller weights to larger concentrations [52,63]. The first step in WLR calculation is to choose the correct *w*_i_. Several authors report that the *w*_i_ should be calculated using Equation (3), where each calibration point is given a weight that is inversely proportional to the corresponding $s_{i}^{2}$ [58,60,62,64].

(3) $$w_{i}=\frac{s_{i}^{-2}}{\sum s_{i}^{-2}/n}$$

where *n* is the number of calibration points i. However, the use of this *w*_i_ requires multiple signal measurements at every calibration point, which can be impractical, costly, and time consuming, as a new calibration curve should be performed every day before analysis. Therefore, other empirical *w*_i_ based on the *x*-variable (i.e., *γ* herein) and *y*-variable (i.e., Δ*i*_p_ herein) can be used and can provide a much simpler approximation of the $s_{i}^{2}$. The most commonly used empirical *w*_i_, also employed in this work, are $\frac{1}{x_{i}}$, $\frac{1}{x_{i}^{0.5}}$, $\frac{1}{x_{i}^{2}}$, $\frac{1}{y_{i}}$, $\frac{1}{y_{i}^{0.5}}$, and $\frac{1}{y_{i}^{2}}$ [49,65,66]. The weighted regression parameters, i.e., weighted slope ($b_{1}^{\left(w\right)})$, weighted intercept ($b_{0}^{\left(w\right)})$, and weighted squared correlation coefficient $\left(R^{2\left(w\right)}\right)$, can thus be calculated using Equations (4)–(6), respectively [52]. The conversion from unweighted OLS parameters to WLR weighted parameters can be performed by considering the *w*_i_ to any Σ and changing *n* to $\sum w_{i}$. Note that, for the OLS method, *w*_i_ is equal to 1 [63].

(4) $$b_{1}^{\left(w\right)}=\frac{\sum w_{i}\cdot \sum w_{i}\cdot x_{i}\cdot y_{i}-\sum w_{i}\cdot x_{i}\cdot \sum w_{i}\cdot y_{i}}{\sum w_{i}\cdot \sum w_{i}\cdot x_{i}^{2}-\sum {\left(w_{i}\cdot x_{i}\right)}^{2}}$$

(5) $$b_{0}^{\left(w\right)}=\frac{\sum w_{i}\cdot x_{i}^{2}\cdot \sum w_{i}\cdot y_{i}-\sum w_{i}\cdot x_{i}\cdot \sum w_{i}\cdot x_{i}\cdot y_{i}}{\sum w_{i}\cdot \sum w_{i}\cdot x_{i}^{2}-\sum {\left(w_{i}\cdot x_{i}\right)}^{2}}$$

(6) $$R^{2\left(w\right)}={\left(\frac{\sum w_{i}\cdot \sum w_{i}\cdot x_{i}\cdot y_{i}-\sum w_{i}\cdot x_{i}\cdot \sum w_{i}\cdot y_{i}}{\sqrt{\sum w_{i}\cdot \sum w_{i}\cdot x_{i}^{2}-\sum {\left(w_{i}\cdot x_{i}\right)}^{2}}\cdot \sqrt{\sum w_{i}\cdot \sum w_{i}\cdot y_{i}^{2}-\sum {\left(w_{i}\cdot y_{i}\right)}^{2}}}\right)}^{2}$$

where $x_{i}$ is *γ* and $y_{i}$ is Δ*i*_p_ at a given calibration point i. The selection of the most appropriate *w*_i_ and consequently the most appropriate weighted model depends on the sum of the absolute relative errors (∑|%RE|), of which %RE is calculated according to Equation (7). The weighted model with the smallest ∑|%RE| is considered the most appropriate [62].

(7) $$\%\mathrm{RE}=\frac{\gamma_{i}^{\mathrm{weighted}\;\mathrm{model}}-\gamma_{i}^{\mathrm{theoretical}}}{\gamma_{i}^{\mathrm{theoretical}}}\cdot 100$$

where $\gamma_{i}^{\mathrm{weighted}\;\mathrm{model}}$ is the *γ* obtained by the weighted model from the measured Δ*i*_p_ at calibration point i and ${}_{i}^{\mathrm{theoretical}}\;$ is the diluted analyte standard concentration at calibration point i. The plot of %RE vs. *γ* in combination with (∑|%RE|) is a useful indicator for evaluating the *w*_i_ in the WLR method [52].

**Figure 3:**
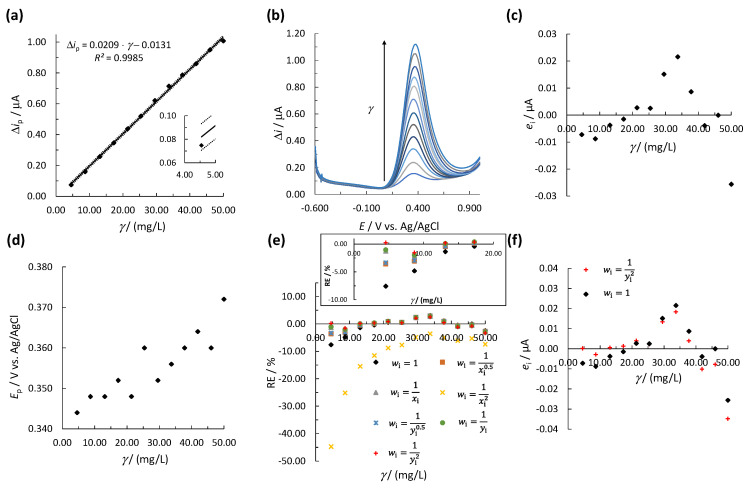
(**a**) The linear calibration curve for UA using the OLS method, where the solid line represents the calibration curve and the dotted lines represent the upper and lower 95% confidence intervals. The insert in (**a**) shows the first calibration point; (**b**) the SWV measurements for constructing the calibration curve; (**c**) the residual analysis, i.e., the plot of *e*_i_ vs. *γ*, (**d**) the change in *E*_p_ vs. *γ*, (**e**) %RE vs. *γ* for the unweighted and weighted models in Table 1 (the insert in (**e**) shows RE at lower concentrations); (**f**) the values of *e*_i_ vs. *γ* for weighted model 7 and unweighted model 1. The measurements were performed by SPCE.

Initially, for UA determination, a new linear calibration curve was constructed using a single measurement for every calibration point (Figure 3a). The corresponding square-wave voltammograms are shown in Figure 3b. The peak potential (*E*_p_) for UA is in the range of 0.344–0.372 V and was shifted to more positive *E* with an increase in *γ* (Figure 3d). The normal distribution of the experimental data was confirmed using the Q-Q plot and the K-S test (see Figure S3 in the Supplementary Material). Since there is only one measurement at every calibration point, a statistical *F*-test to evaluate heteroscedasticity was not possible. Therefore, visual inspection by residual analysis was performed. Figure 3c shows that the *e*_i_ form a non-random inverted v-shaped distribution around the *γ*-axis, indicating the heteroscedasticity of the data. Moreover, even though the first calibration point was within the required 95% confidence interval, a large deviation from the calibration curve was observed; thus, a lower accuracy at low UA concentrations was expected (insert in Figure 3a). Based thereon, the WLR method should be employed instead of the OLR method. Therefore, for each empirical *w*_i_ mentioned above, the weighted regression parameters and ∑|%RE| were calculated for both analytes; the results for UA and EP are shown in Table 1 and Table S1 (Supplementary Material), respectively. The generated weighted models were compared by means of ∑|%RE|. The best weighted model was also compared with an unweighted model (i.e., the regression parameters, *b*_1_, *b*_0_, and *R^2^*, were calculated using the OLS method by Equations (4)–(6), *w*_i_ = 1). The lowest ∑|%RE| was determined for weighted model 7 with $w_{i}=\frac{1}{y_{i}^{2}}$ (Figure 3e and Table 1). The obtained ∑|%RE| for model 7 was much lower than ∑|%RE| calculated using unweighted model 1 (Table 1). The plot of %RE vs. *γ* in Figure 3e shows that %RE were much lower at lower concentrations for model 7 compared to model 1. Therefore, model 7 should provide a more accurate result at lower concentrations of the linear concentration range. Moreover, the residual analysis showed that *e*_i_ were much lower at the first four calibration points for model 7 compared to model 1 (Figure 3f). Based on the above, model 7 with $w_{i}=\frac{1}{y_{i}^{2}}$ was used to validate the accuracy and precision of the WLR method for UA.

The same procedure for determining the best *w*_i_ and the best weighted model was used for EP. The results are shown in Figure S4 and Table S1 (in the Supplementary Material). The best weighted model for EP determination was again model 7 with $w_{i}=\frac{1}{y_{i}^{2}}$; thus, model 7 was used to validate the accuracy and precision of the WLR method for EP.

### 3.3. Accuracy and Precision

The accuracy and precision of both electroanalytical methods were tested at the lowest, middle, and highest concentration levels of the linear concentration range. Three measurements were performed at each level. The measured Δ*i*_p_ were checked for possible outliers using Dixon’s and Grubb’s tests. However, no outliers were detected. The accuracy was evaluated by calculating the average recovery value. The method was deemed accurate if the average recovery value was between 80.00% and 120.00% [67]. Precision was assessed using the relative standard deviation (RSD), which had to be equal to or less than 20.00% for a method to be considered precise [67]. Accuracy and precision were tested using single-drop analysis, as described in Section 2.4. Quantification for both analytes was performed using the WLR calibration curves, as presented in Section 3.2 (both using model 7 with $w_{i}=\frac{1}{y_{i}^{2}}$, Table 1). Since the heteroscedastic behavior of the experimental data can have a significant impact on the accuracy at lower concentrations of the analytes in the linear concentration range, a comparison between the results obtained with the best weighted model, i.e., model 7 (WLR method), and unweighted model 1 (OLS method) is described to justify the use of the WLR method. The obtained results for UA and EP are shown in Table 2. In addition, the results obtained with the OLS and WLR methods were also evaluated using a two-sample *t*-test for equal means. On that basis, *t*^exp^ was calculated from the results obtained for each concentration level tested and compared with *t*^crit^ (where *t*^crit^ is a critical value at 95% confidence and four degrees of freedom, (*n*_1_ + *n*_2_ − 2 = 4 as *n*_1_ = 3 and *n*_2_ = 3)). In general, statistical difference is considered significant for the two obtained average recovery values using the OLS and WLR methods when *t*^exp^ > *t*^crit^ [68].

Table 2 shows that, when the WLR method was employed for UA and EP, the obtained results representing accuracy (the average recovery value) and precision (RSD) were within the required limits, i.e., a recovery interval of 80.00–120.00% and RSD < 20.00% [67], for all three concentrations tested using both the OLR and WLR methods.

On the other hand, a statistically significant difference of the obtained results using the WLR and OLR methods is present for the lowest UA and EP concentrations tested (i.e., 6.14 mg/L for UA and 5.59 mg/L for EP). The accuracy was significantly better for the WLR method (see the average recovery values in Table 2). In addition, a statistical difference between the average recovery values for the OLS and WRL methods is observed for UA and EP at the lowest concentration level, when *t*^exp^ > *t*^crit^. The latter justifies the use of the WLR method instead of OLR.

At the middle (25.00 mg/L for UA and 50.25 mg/L for EP) and highest (47.62 mg/L for UA and 97.56 mg/L for EP) concentration levels tested, the WLR method did not significantly improve the analytical results. The latter was also confirmed by the two-sample *t*-test as *t*^exp^ < *t*^crit^. Based on the above, we can confirm that the use of the WLR method improved the accuracy at lower concentrations for both analytes as expected, and is usually the main reason for employing the WLR method.

### 3.4. Robustness

According to The International Council for Harmonization of Technical Requirements for Pharmaceuticals for Human Use, the robustness of an analytical method is intended to show the reliability of an analysis with respect to deliberate variations in method parameters. An example of typical variations in the case of liquid chromatography is different columns, provided by a different supplier and/or columns with different lots [56]. For example, the robustness of the method was tested using twelve different columns [69]. Herein, the robustness of the developed electroanalytical methods for the determination of UA and EP was tested by employing SPE sensors from another manufacturer, and it was evaluated in terms of accuracy and precision under the same operating conditions. DRP-110 sensors from the manufacturer DropSens were used for this purpose. To find the best *w*_i_ and weighted model for D-SPCE, the procedure described in Section 3.2 was performed. The results are shown in Table 3.

The determination of accuracy and precision was performed as described in Section 3.3. The best weighted model for both methods was determined to be model 7 with *w*_i_ = $\frac{1}{y_{i}^{2}}$. Therefore, model 7 was selected for the accuracy and precision determination of EP and UA using D-SPCE. The results were evaluated using the OLS and WLR methods to confirm the necessity of the WLR method for UA and EP determination using single-drop analysis (as was the case for SPCE, presented above). The results in terms of accuracy and precision obtained with unweighted model 1 (OLS method) and the best weighted model, i.e., model 7 (WLR method), are shown in Table 4.

The measurements obtained at three different concentration levels were considered accurate and precise, as the average recovery values were between 80.00% and 120.00% and the RSD values for both methods were less than 20.00% when the WLR method was used (Table 4).

No significant difference was observed between the results evaluated using the OLS and WLR methods for the determination of UA at all three tested levels (*t*^exp^ < *t*^crit^). On the other hand, when the OLS method was used for the determination of EP, the average recovery value at the lowest concentration was below the required lower average recovery limit of 80.00% [67], meaning that the measurement at a concentration of 5.69 mg/L was inaccurate. By using the WLR method, the average recovery value at the same concentration increased to 83.27%, which was within the required average recovery interval of 80.00–120.00%. At the middle and highest concentration levels, no significant difference was found between the results evaluated by the OLS and WLR methods. Therefore, it can be concluded that the use of the WLR method is also mandatory for the determination of EP using single-drop analysis on D-SPCE (as was the case for SPCE, as presented above).

Since the D-SPCE sensors provided accurate and precise results under the same operating conditions as the SPCE sensors, the methods for the determination of EP and UA were considered robust for the determination of UA and EP.

## 4. Real Sample Analysis of EP and UA

The validated electroanalytical methods presented above were used for real sample analysis using single-drop analysis and SPCE. The content of UA and EP in human urine and an EP auto-injector, respectively, were determined. Before analysis, the calibration curves were constructed for both WLR methods using the best weighted model, i.e., model 7 (Table 1), for both real samples. To test the accuracy and precision of the obtained results, the EP from an EP auto-injector and human urine were spiked with a solution of a known amount of EP and UA standard, respectively. The criteria for accuracy and precision are given in Section 3.3. For UA determination, urine samples were collected from three members of our laboratory in the morning and analyzed on the same day. The urine samples were then diluted to the required concentration without any sample pretreatment. A small volume of EP from an EP auto-injector (300 µL) was transferred to a beaker. Since the declared concentration of EP in EP auto-injector was 150 µg/0.3 mL, which was well above linear concentration range, the real sample must be diluted to the required concentration (which was within linear concentration range). All measurements were performed three times, and the average results of the real sample analysis are shown in Table 5 (one of three real sample analyses is reported for UA, while the other two real samples for UA determination had Re within the 80.00–120.00% limits and RSD below the 20.00% limit, as required).

The determined concentrations of UA and EP in the real samples (Table 5) were accurate and precise, as the average recovery values were within the 80.00–120.00% limits and the RSD values were well below the 20.00% limit. The determined concentration of EP corresponds to the declared value and the concentration of UA corresponds to the expected value in human urine [70].

## 5. Conclusions

The focus of this work was on developing and validating electroanalytical methods for the determination of UA and EP in a low volume of sample, i.e., a single drop of 50 µL, which was pipetted directly on to the surface of a screen-printed carbon electrode (SPCE) using weighted linear regression (WLR). Square-wave voltammetry (SWV) was employed for the analysis. To establish the proper functionality of the SPCE and D-SPCE sensors, the reversibility of the diffusion-controlled reaction of the potassium hexacyanoferrate(III) system was tested using cyclic voltammetry and single-drop analysis. All sensors used in this work met the criteria for a reversible diffusion-controlled reaction. The limit of detection (LOD), limit of quantification (LOQ), linearity, accuracy, precision, and robustness were tested as part of the validation.

For UA, the obtained LOD was 1.00 mg/L and the obtained LOQ was 2.26 mg/L. The determined LOD and LOQ for EP were 1.20 and 2.01 mg/L, respectively. The determined linear concentration ranges for UA and EP were 4.55–50.00 mg/L and 3.87–100.49 mg/L, respectively. The normal distribution of the experimental data was checked using the quantile-quantile plot and the Kolmogorov-Smirnov test. The latter was confirmed in all cases. Since all requirements for using ordinary least squares (OLS) regression to estimate linearity were not met, the WLR method was employed instead. One of the requirements is that the data are homoscedastic. Therefore, the homoscedastic behavior of the experimental data was visually tested by a residual analysis and by a statistical *F*-test. The *e*_i_ showed a non-random distribution around the concentration axis for both analytes, indicating heteroscedasticity. A statistical *F*-test showed that the $s_{i}^{2}$ of the response over the entire calibration range were statistically different as *F*^exp^ > *F*^crit^ for UA and EP, confirming heteroscedasticity. In order to find the best weighted model for both methods, new calibration curves were constructed for both analytes, and the following empirical weighting factors (*w*_i_) were tested: $\frac{1}{x_{i}}$, $\frac{1}{x_{i}^{0.5}}$, $\frac{1}{x_{i}^{2}}$, $\frac{1}{y_{i}}$, $\frac{1}{y_{i}^{0.5}}$, and $\frac{1}{y_{i}^{2}}$. The *w*_i_ considered the most appropriate to be the one with the lowest sum of absolute relative percentage errors at every calibration point (∑|%RE|). The *w*_i_ with the lowest ∑|%RE| was $\frac{1}{y_{i}^{2}}$ for both analytes. The selected best weighted model was used to validate the accuracy and precision of the UA and EP methods. Furthermore, a comparison between the results obtained with the OLS and WLR methods was carried out using a two-sample *t*-test for equal means to show the necessity of using the WLR method for UA and EP analyses. The accuracy and precision were tested at the lowest, middle, and highest concentration levels of the linear calibration range. The results of both methods obtained with the WLR method were accurate and precise, with average recovery values in the range of 99.52–108.18% and relative standard deviations in the range of 0.15–1.57%, both of which were within the required limits. Moreover, the WLR method improved the analytical results at the lowest concentrations of the linear calibration range in terms of precision and accuracy. The robustness of the two analytical methods was tested by employing SPE sensors from another manufacturer. The D-SPCE sensor provided accurate and precise results obtained under the same operating conditions as the SPCE sensors. Thus, both methods are considered robust. The real sample analysis of UA and EP was performed using the best weighted model. The content of UA in human urine and EP in an EP auto-injector was tested. The average recovery values were 94.35% (RSD = 5.65%) and 101.01% (RSD = 2.75%) for UA and EP, respectively. Thus, both methods showed accurate and precise results for the real sample analysis.

The developed and validated methods for UA and EP determination are a great substitute for chromatographic techniques in terms of the speed and cost of analysis. Single-drop analysis enables analysis at very low sample volumes and on-site analysis. In addition, no conventional electrochemical cell and stirrer are required, increasing the miniaturization of the system.

## Figures and Tables

**Table 1:** The empirical *w*_i_ with corresponding calculated weighted regression parameters ($b_{1}^{\left(w\right)}$, $b_{0}^{\left(w\right)}$, $R^{2(w)}$) and ∑|%RE| for UA. The *w*_i_ = 1 represents the unweighted model.

			SPCE			
Analyte	Model No.	$w_{i}$	$b_{1}^{\left(w\right)}$	$b_{0}^{\left(w\right)}$	$R^{2\left(w\right)}$	∑|%RE|
UA	1 (unweighted model)	1	0.0209	−0.0131	0.9985	24.74
	2	$\frac{1}{x_{i}^{0.5}}$	0.0211	−0.0176	0.9989	18.62
	3	$\frac{1}{x_{i}}$	0.0212	−0.0203	0.9992	15.43
	4	$\frac{1}{x_{i}^{2}}$	0.0213	−0.0223	0.9995	145.41
	5	$\frac{1}{y_{i}^{0.5}}$	0.0211	−0.0178	0.9990	18.32
	6	$\frac{1}{y_{i}}$	0.0212	−0.0205	0.9993	15.31
	7	$\frac{1}{y_{i}^{2}}$	0.0213	−0.0222	0.9995	14.02

**Table 2:** The average recovery and RSD values for UA and EP, obtained using unweighted model 1 (OLS method) and weighted model 7 (WLR method). The measurements were performed by SPCE.

SPCE							
		OLS (Unweighted Model 1)			WLR (Weighted Model 7)		
	*γ*/(mg/L) (Theoretical)	*γ*/(mg/L) (Determined)	Average Recovery/%	RSD/% (n = 3)	*γ*/(mg/L) (Determined)	Average Recovery/%	RSD/% (n = 3)
UA	6.14	5.78	94.18	1.08	6.11	99.52	1.00
	25.00	25.51	102.04	1.60	25.50	102.00	1.57
	47.62	47.79	100.37	0.80	47.40	99.54	0.79
EP	5.69	6.69	117.64	0.91	5.70	100.20	1.10
	50.25	49.84	99.20	0.46	50.22	99.95	0.47
	97.56	103.46	106.04	0.15	105.54	108.18	0.15

**Table 3:** A comparison between unweighted model 1 (OLS method) and the best weighted model, i.e., model 7 (WLR method), with weighted regression parameters and ∑|%RE| for UA and EP.

			D-SPCE			
Analyte	Model No.	$w_{i}$	$b_{1}^{\left(w\right)}$	$b_{0}^{\left(w\right)}$	$R^{2\left(w\right)}$	∑|%RE|
UA	1 (unweighted model)	1	0.4351	0.0250	1.0000	5.90
	7	$\frac{1}{y_{i}^{2}}$	0.4369	−0.0184	1.0000	4.03
EP	1 (unweighted model)	1	0.2681	0.1295	0.9990	39.15
	7	$\frac{1}{y_{i}^{2}}$	0.2727	−0.0912	0.9994	20.65

**Table 4:** Average recovery and RSD values for UA and EP, calculated with unweighted model 1 (OLS method) and weighted model 7 (WLR method), obtained by D-SPCE.

	D-SPCE						
		OLS (Unweighted Model 1)			WLR (Weighted Model 7)		
	*γ*/(mg/L) (Theoretical)	*γ*/(mg/L) (Determined)	Recovery/%	RSD/%	*γ*/(mg/L) (Determined)	Recovery/%	RSD/%
UA	6.14	6.29	102.47	1.63	6.36	103.65	1.60
	25.00	24.13	96.53	0.40	24.13	96.51	0.39
	47.62	44.81	94.10	1.10	44.72	93.91	1.09
EP	5.69	3.99	70.23	6.71	4.74	83.27	5.57
	50.25	53.22	105.93	3.15	53.13	105.75	3.11
	97.56	97.78	100.22	2.39	96.93	99.35	2.37

**Table 5:** Real sample analysis results for UA and EP obtained using the best weighted model. All values are given as the average of three measurements.

	* *γ*/(mg/L) (Initially Determined)	γ/(mg/L) (Spiked)	** *γ*/(mg/L) (Determined)	Average Recovery/%	RSD/%	*** *γ*/(mg/L) (Sample Content)
UA in human urine	6.33	25.00	29.90	94.35	5.65	316.29
EP in an auto-injector	23.92	50.52	74.95	101.01	2.75	478.36

1. * Determined *γ* of the diluted real sample. 2. ** Determined *γ* after spiking the real sample with the solution of the corresponding analyte standard. 3. *** Calculated *γ* by taking into account the dilution factor of the real sample.

## Data Availability

The data presented in this study are available on request. The data are not publicly available due to technical limitations.

## Supplementary Materials

The following are available online at https://www.mdpi.com/article/10.3390/bios11080285/s1, Figure S1: An example of a properly functioning SPCE; (a) cyclic voltammograms of 1.0 M KCl containing 10 mM K_3_Fe[CN]_6_, measured at the following *ν*: 10, 20, 50, 75, 125, 150, 175, and 200 mV/s, (b) *i*_pa_ vs. $\sqrt{v}$, and *i*_pc_ vs. $\sqrt{v}$. Figure S2: (a,c) Quantile-quantile (Q-Q) plots and (b,d) statistical Kolmogorov-Smirnov (K-S) test plots confirming the normal distribution of y-direction errors for the first set of calibration curves that were obtained with the average response of three replicate measurements at every calibration point for (a,b) uric acid (UA) and (c,d) epinephrine (EP). The z_theoretical_ represents the z-value of the standard normal distribution, whereas z_actual_ is the actual z-value calculated based on the obtained experimental data. F_E_ and F_O_ stand for the expected and observed frequency, respectively. F_Oi_ and F_Oi–1_ represent F_O_ for the i/n and i–1/n (i = 1, 2, …, n) calibration points, respectively. Figure S3: (a,c) Q-Q plots and (b,d) statistical K-S test plots confirming the normal distribution of y-direction errors for the second set of the freshly obtained calibration curves (one measurement at every calibration point) that were used for the weighted linear regression; (a,b) UA and (c,d) EP. Figure S4: (a) The linear calibration curve for EP using the OLS method, where the solid line represents the calibration curve and the dotted lines represent the upper and lower 95% confidence interval limits. The insert in Figure S4a shows the first calibration point, (b) the SWV measurements to construct the calibration curve, (c) the residual analysis, i.e., the plot of *e*_i_ vs. *g*, (d) the change of peak potential (*E*_p_) vs. *g*, (e) %RE vs. *g* for the unweighted and weighted models in Table S1 (the insert in Figure S4e shows RE at lower concentrations), (f) the values of *e*_i_ vs. γ for weighted model 7 and unweighted model 1. The measurements were performed with SPCE. Table S1: The empirical weighting factors (*w*_i_) with the corresponding calculated weighted regression parameters (weighted slope $\left(b_{1}^{\left(w\right)}\right)$, weighted intercept ($b_{0}^{\left(w\right)})$, weighted squared correlation coefficient ($R^{2\left(w\right)})$) and the sum of absolute relative errors (∑|%RE|) for EP. The unweighted model is marked *w*_i_ = 1.
