# Supplementary material for: Single-Drop Analysis of Epinephrine and Uric Acid on a Screen-Printed Carbon Electrode
Source: Biosensors (Basel). 2021 Aug 19;11(8):285. doi: 10.3390/bios11080285 (PMC8392267; doi:10.3390/bios11080285)
Supplement: Supplementary file 1 [file biosensors-11-00285-s001.zip › biosensors-1330084-supplementary.pdf]

SUPPLEMENTARY MATERIAL

Single-drop analysis of epinephrine and uric acid on a screen-printed carbon electrode

David Majer[1], Matjaž Finšgar[1,*]
[1]University of Maribor, Faculty of Chemistry and Chemical Engineering, Smetanova ulica 17, 2000 Maribor, Slovenia

1. The reversibility and diffusion-controlled reaction of potassium hexacyanoferrate(III) using cyclic voltammetry

The criteria for the reversible diffusion-controlled oxidation/reduction reaction of potassium hexacyanoferrate(III) ($K_3Fe[CN]_6$) are described as follows: i) the anodic (i_{pa}) and cathodic (i_{pc}) peak currents should show a linear relationship with the square root of the scan rate ($\sqrt{\nu}$) for the diffusion-controlled reaction of $K_3Fe[CN]_6$, ii) the absolute value of the i_{pa} and i_{pc} ratio should be equal to 1.00 for a reversible reaction, iii) the difference between the anodic (E_{pa}) and cathodic (E_{pc}) peak potential should be equal to 59 mV/n_e (where n_e is the number of exchanged electrons in the redox reaction, and for the $K_3Fe[CN]_6$ system n_e = 1) for a reversible reaction, iv) E_{pa} and E_{pc} should not change with increasing ν [1].

An example of a properly functioning screen-printed carbon electrode (SPCE) that provided the results for the reversible diffusion-controlled reaction of $K_3Fe[CN]_6$ that were sufficient to use an SPCE for further analysis is shown in Figure S1.

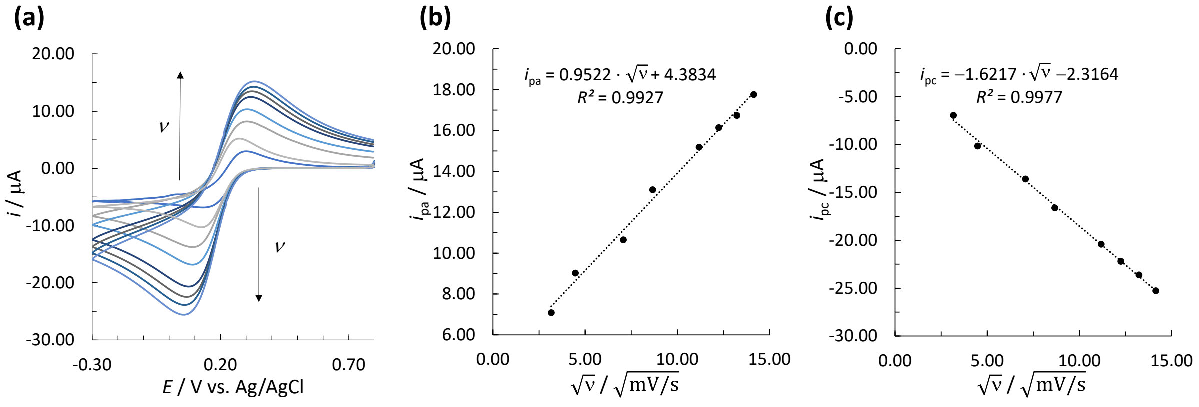

Figure S1: An example of a properly functioning SPCE; a) cyclic voltammograms of 1.0 M KCl containing 10 mM $K_3Fe[CN]_6$, measured at the following ν: 10, 20, 50, 75, 125, 150, 175, and 200 mV/s, b) i_{pa} vs. $\sqrt{\nu}$, and i_{pc} vs. $\sqrt{\nu}$.

Figure S1a shows cyclic voltammograms where i_{pa} and i_{pc} increased with ν. Furthermore, i_{pa} and i_{pc} showed a linear relationship with $\sqrt{\nu}$, thus satisfying criteria i) (Figure S1b,c). The ratio between i_{pa} and i_{pc} was 1.02 at a ν of 10 mV/s. The ratio then decreased with increasing ν, indicating a quasi-reversible system. The difference between E_{pa} and E_{pc} at a ν of 10 mV/s was 167 mV. The potential (E) difference then increased with increasing ν. The E_{pa} and E_{pc} slightly shifted towards more positive and more negative E with increasing ν, respectively. Despite small deviations from the ideal criteria ii)-iv), the SPCE satisfies all four criteria and is suitable for further analysis.

* Corresponding author
Address: University of Maribor, Faculty of Chemistry and Chemical Engineering, Smetanova ulica 17, 2000 Maribor, Slovenia
e-mail: matjaz.finsgar@um.si; *phone*: +386 2 2294 447

2. The Q-Q plots and Kolmogorov-Smirnov test plots for the normal distribution

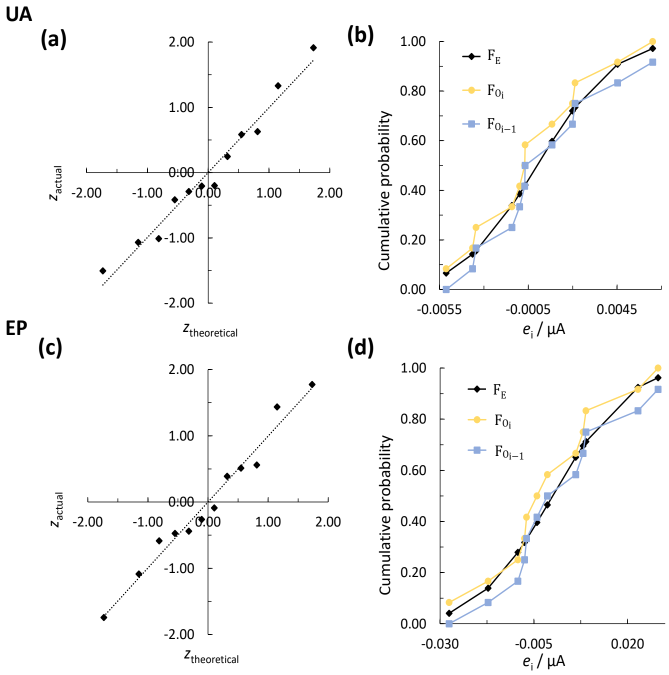

Figure S2: a,c) Quantile-quantile (Q-Q) plots and b,d) statistical Kolmogorov-Smirnov (K-S) test plots confirming the normal distribution of y-direction errors for the first set of calibration curves that were obtained with the average response of three replicate measurements at every calibration point for a,b) uric acid (UA) and c,d) epinephrine (EP). The $z_{theoretical}$ represents the z-value of the standard normal distribution, whereas z_{actual} is the actual z-value calculated based on the obtained experimental data. F_E and F_O stand for the expected and observed frequency, respectively. F_{Oi} and F_{Oi-1} represent F_O for the i/n and i–1/n (i = 1, 2, ..., n) calibration points, respectively [2].

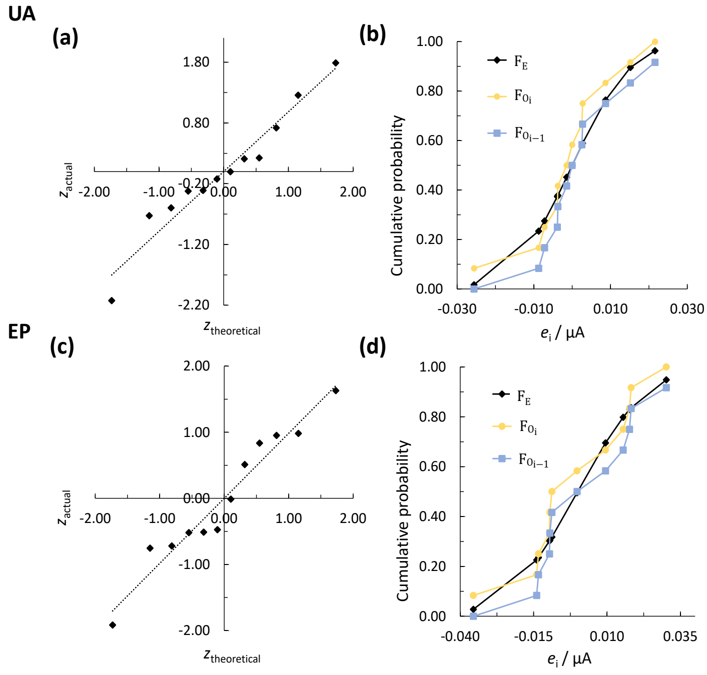

Figure S3: a,c) Q-Q plots and b,d) statistical K-S test plots confirming the normal distribution of y-direction errors for the second set of the freshly obtained calibration curves (one measurement at every calibration point) that were used for the weighted linear regression; a,b) UA and c,d) EP.

3. Weighted linear regression results for epinephrine

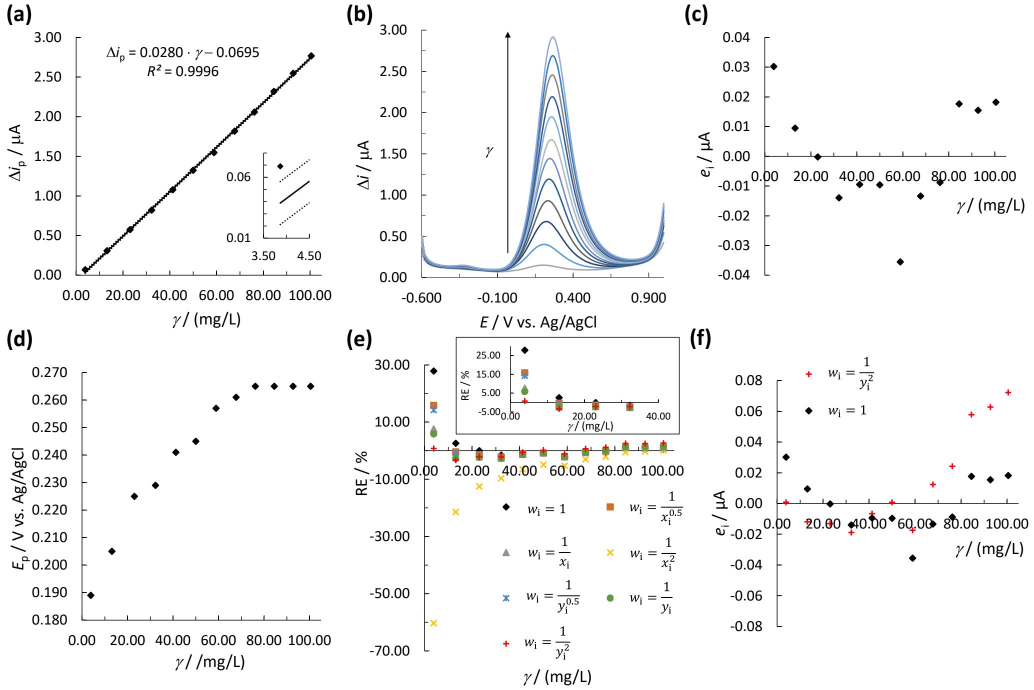

Figure S4: a) The linear calibration curve for EP using the OLS method, where the solid line represents the calibration curve and the dotted lines represent the upper and lower 95% confidence interval limits. The insert in Figure S4a shows the first calibration point, b) the SWV measurements to construct the calibration curve, c) the residual analysis, i.e. the plot of e_i vs. γ, d) the change of peak potential (E_p) vs. γ, e) %RE vs. γ for the unweighted and weighted models in Table S1 (the insert in Figure S4e shows RE at lower concentrations), f) the values of e_i vs. γ for weighted model 7 and unweighted model 1. The measurements were performed with SPCE.

Table S1: The empirical weighting factors (w_i) with the corresponding calculated weighted regression parameters (weighted slope ($b_1^{(w)}$), weighted intercept ($b_0^{(w)}$), weighted squared correlation coefficient ($R^{2(w)}$)) and the sum of absolute relative errors ($\sum|\%\mathrm{RE}|$) for EP. The unweighted model is marked w_i=1.

SPCE						
**Analyte**	**Model No.**	w_i	$b_1^{(w)}$	$b_0^{(w)}$	$R^{2(w)}$	$\sum\|\%\mathrm{RE}\|$
EP	1 (unweighted model)	1	0.0280	−0.0695	0.9996	38.75
	2	$\frac{1}{x_i^{0.5}}$	0.0278	−0.0554	0.9996	27.88
	3	$\frac{1}{x_i}$	0.0276	−0.0458	0.9996	23.39
	4	$\frac{1}{x_i^2}$	0.0272	−0.0377	0.9995	126.71
	5	$\frac{1}{y_i^{0.5}}$	0.0278	−0.0536	0.9996	27.13
	6	$\frac{1}{y_i}$	0.0276	−0.0437	0.9996	22.50
	7	$\frac{1}{y_i^2}$	0.0272	−0.0368	0.9995	19.55

4. References

1. Finšgar, M.; Xhanari, K.; Otmačić Ćurković, H. Cyclic Voltammetry as an Electroanalytical Tool for Analysing the Reaction Mechanisms of Copper in Chloride Solution Containing Different Azole Compounds. *Current Analytical Chemistry* **2020**, *16*, 465-474, doi:http://dx.doi.org/10.2174/1573411014666180704114202.
2. Massart, D.L.; Vandeginste, B.G.; Buydens, L.M.C.; Lewi, P.J.; Smeyers-Verbeke, J.; Jong, S.D. *Handbook of Chemometrics and Qualimetrics: Part A*; Elsevier Science Inc.: 1997.
